# Observations on the Various Types of Fever and Dysentery, Made in the Wards of the Illinois General Hospital, during the Six Months Ending Sept. 25, 1852

**Published:** 1852-10

**Authors:** N. S. Davis

**Affiliations:** Physician to the Hospital, and Prof. of Practical Medicine and Pathology in Rush Med. College; Chicago


ART. II.—*Observations on the Various Types of Fever and Dysentery made in the Wards of the Illinois General Hospital, during the Six Months ending Sept.* 25, 1852. By N. S. DAVIS, M. D., Physician to the Hospital, and Prof. of Practical Medicine and Pathology in Rush Med. College.

THE treatment of fevers and dysentery constitutes so important a part of the daily duties of the physician, that it can scarcely receive too much of our attention; and I hold it to be more especially the duty of those who occupy places in connection with public hospitals, in which greater facilities are afforded for comparative observations than in private practice, to give to the profession the results of their experience. With this view I have caused accurate records to be kept, not only of each case, but of each day's prescriptions, during the whole period of my connection with the Illinois General Hospital.

During the last term of service, there were admitted and treated in the institution 41 cases of intermittent and remittent fevers; 24 of typhus and typhoid fevers; and 12 of dysentery.

Of the first class all recovered and were discharged well; of the second, 21 recovered and 3 died; and of the third, 9 recovered, 2 died and one was removed by friends before its termination either in recovery or death. It may be well to remark that most of the foregoing patients belonged to the poorer class of society, and were seldom brought to the hospital until one, two, or three weeks after the commencement of the attack.

A large majority of the intermittents were chronic cases, which had been suffered to continue until that anæmic state had supervened, marked by muscular weakness, paleness of pro-labia and tongue, sallowness of skin, and in several instances distinct enlargement of the liver or spleen, or both.

In those cases not complicated with visceral enlargements or inflammation, the treatment was generally commenced with the following prescription, viz.:

Sulph. quinine, 15 grs.
Pulv. opii, 3 grs. mix, divide into three doses, and take one every three hours, commencing at such time that the last dose will be taken about one hour before the expected chill. This was generally sufficient to interrupt the paroxysms, and if so, the continuance of the sulph. quinine in doses of two grains combined with ten or fifteen grains of chloride of sodium, and given three times a-day for two or three days, and once a-day for a week longer, very certainly prevented relapse and rapidly restored the patient. Where the anæmia is well marked, I often add from three to five grains of carb. iron to each dose of the quinine and salt. In several chronic cases, where the patients were much debilitated, the pulse soft, slow and easily compressed, and the organic actions generally sluggish or torpid; instead of giving quinine and opium, my first prescription has been sulph. quinine, 10 grs.; chloride sodium, ℨjss, mix and divide into four powders; one of which is given every three hours, at such time that the last one will be taken one hour before the expected paroxysm. This has interrupted the paroxysms in such cases with even greater certainty than the quinine and opium.

The subsequent treatment has been the same as already indicated. Where visceral congestions and enlargements exist, I generally make no other alteration in the treatment than to add to the first few doses of quinine given, from two to five grains of blue mass or its equivalent of calomel, and follow it in twenty-four hours by a laxative of rhubarb and soda, or castor oil and oil of turpentine, and a blister over the affected organ. All active purging is avoided; costiveness being obviated by the moderate use of the laxatives just named, or by enemas of warm water and common salt.

Very few cases of remittent fever have come into the hospital the first three or four days after the commencement of the disease. Where such has been the case, and the febrile exacerbations well marked; the pulse full and active; the skin dry and hot; the tongue covered with a white or yellowish white fur; the head, back, and limbs painful; and all the internal secretions scanty; the treatment has been commenced by giving, at intervals of two or three hours, a powder composed of pulv. opii, from 1 gr. to 2 grs.; calomel, 3 grs.; sup. carb. soda, 4 grs.; alternated with a tea-spoonful of spts. nit. dulc. After three or four of these powders have been given, the bowels are freely opened by the exhibition of castor oil, ℥ss, oil turpentine, ʒj, mixed. The operation of the oils is immediately followed by the exhibition of sulph. quinine and opium, in doses of four grains of the former to one or two grains of the latter; repeated every three or four hours until four doses have been taken. The immediate effect of this treatment is, to procure from the bowels two or three copious, dark, and offensive evacuations, followed by a removal of the thirst, pains in the head and back, and an increased secretion from the skin and kidneys. In recent cases, the disease is thus arrested entirely during the first 48 hours of treatment; after which the case may be treated in all respects the same as already mentioned in reference to intermittents after the paroxysms have been arrested. Beef tea or other animal broth, pretty well salted, should constitute an important part of the diet, and the bowels should be carefully regulated, if possible, without the use of active cathartics. But a large proportion of the cases of remittent fever received into the hospital, like those of intermittents, have already been sick one or two weeks, and have taken emetics or purgatives, and sometimes both.

In a large majority of these cases, I find the skin dry and harsh; the tongue red on the tip and edges, with a dry dirty fur along the middle and back part; the head and back moderately painful; the pulse quick, soft, and easily compressed; the mind often wandering during the height of the febrile exacerbation; the bowels sometimes moderately tympanitic with slight tenderness to firm pressure, or they are very flaccid and empty; the urine is scanty and high colored; the fæcal evacuations thin, dark brown, numbering from two to six in twenty-four hours; and the febrile remissions imperfect. In such cases I have occasionally found full doses of quinine and opium to operate favorably, allaying the manifest irritation of the mucous surfaces, and inducing copious diaphoresis; but in far the larger number of instances they serve only to increase the dullness and tendency to delirium, without improving any of the symptoms; while calomel and cathartics aggravate the intestinal irritation without the slightest control of the fever. During the last six months I have uniformly commenced the treatment of such cases in the following manner, viz.:

℞ Oil turpentine, ʒij

Acetum opii, ʒij

Gum Arabic, White sugar, } *a a* ʒiij rub together thoroughly

And add Water, ℥ij mix, give one tea-spoonful every three or four hours, with three grains sulph. quinine, added to each. If the patient does not bear well the quinine, or if the pulse be quite feeble with more frequent evacuations from the bowels, I give the emulsion without the quinine, and between each dose give fifteen or twenty grains of chloride sodium, combined with one grain of pulverized opium. This with the regular administration of animal broth for nourishment, and occasional blisters on the abdomen or chest, as the signs of local disease may indicate, generally produces a rapid improvement in all the symptoms; and by continuing the same medicine at longer intervals, with more liberal diet, the cure will be complete in from one to three weeks. In all these cases there are two important sources of danger. The first consists in depressing the elementary vital forces too low, and thereby inducing a true typhoid condition of the system. The second arises from the development of local complications, of which inflammation and ulceration of the mucous membrane of the intestines is by far the most insidious and frequent in summer, and pneumonia and bronchitis in winter. And the practitioner cannot study with too much patience and accuracy the indications or symptoms that point early to the development of either of these results.

I have already dwelt longer, however, on this part of my subject than I intended, my chief object being to comment on the more continued forms of fever and dysentery.

Of the 24 cases of continued fever embraced in this report, eight were well marked cases of typhus, and the remaining sixteen typhoid or mixed. In making this distinction, I have followed the example of most writers of the present day, without being satisfied of its practical utility or correctness. For after all the discussions on the subject, the *diagnostic* symptoms which serve to distinguish the one variety from the other, are so indefinite and unsatisfactory that the most enlightened practitioner is often wholly unable to satisfy himself under which head a given case ought to be placed. Hence, of the one hundred cases of continued fevers mentioned by Prof. Austin Flint, in his recent volume of Clinical Reports, almost as many are left in the list of *doubtful* as are unhesitatingly placed in the list of typhus or typhoid cases. Such a fact is of itself sufficient evidence that there are no generally recognized and plain differences, which serve to distinguish the one from the other. Hence, it would seem to me much better either to regard the two as different phases or degrees of the same general morbid state, or to unite with certain foreign writers in calling all cases typhoid in which signs of intestinal irritation or ulceration were manifest, and all not presenting this complication, typhus.

Of the twenty-four cases now under consideration, sixteen were Irish, five German, and three Norwegian or Swedish. Ten presented the characteristic typhus eruption over the abdomen and chest; sixteen presented evidence of disease of the mucous surface of the intestines, such as liquid and offensive discharges more frequent than natural, a full and more or less tympanitic state of the abdomen, with some tenderness to firm pressure; and in all wandering of mind was present at some period of the disease, generally at night, and characterized by a great disposition to get out of bed. In six cases there were distinct signs of pneumonia, almost always involving the middle and inferior lobes; while bronchitis was plainly present in only four cases. Of the three fatal cases, one was admitted in March, with the middle and lower lobe of one lung in a state of hepatization with bloody expectoration, and death took place on the eighth day after admission. The second was a German, admitted in an advanced stage of disease, with pneumonic inflammation in the inferior lobes of both lungs, and a very troublesome epistaxis; he died on the sixth day after admission. The third case was an Irish woman, who had been sick four weeks before admission. She presented a strongly marked typhoid general condition with bronchial and intestinal complications; there was loud mucous ronchus over the upper and central portions of both lungs, with harassing cough and expectoration of a dirty-colored muco-purulent matter; the tongue was brown and dry in the centre, the abdomen full and tympanitic, and the intestinal evacuations thin, brown, copious, and numbering three or four every twenty-four hours. She died on the fifth day after admission. A *post-mortem* examination revealed extensive ulceration of the mucous membrane of the ilium, with several small isolated ulcers in the mucous surface of the colon; and well marked inflammation and thickening of the mucous lining of the bronchial tubes. There was also a calcareous deposit, equal in size to a large pea, attached to one of the larger bronchial ramifications in the left lung. The posterior and lower parts of the lungs were engorged with dark blood, but not inflamed.

Instead of stating my views of the treatment of typhoid and typhus cases, I have thought it might be more satisfactory to the reader to copy from the hospital note-book, kept by my valued assistants, Drs. Parker and Miller, the exact details of one or two cases.

Mr. M., an Irish laborer, aged about thirty-five years, was admitted on the 14th of the month. He had been sick about seven days with the ordinary symptoms of continued fever. He had been visited by a judicious physician, who had directed him three or four powders, each composed of calomel, 2 grs., and pulv. Doveri 6 grs., given at intervals of four hours, and followed by a table-spoonful of castor oil, which had opened the bowels freely but not excessively.

At the time of admission, he presented a dull, heavy expression of countenance, slight redness of the conjunctiva; the tongue was covered with a brownish fur, and moderately dry; the skin dry and hot; the abdomen full, but not tympanitic; the pulse 110, full, but soft and easily compressed; the skin over the abdomen and chest thickly covered with a small red rash or eruption; the urine nearly natural; the alvine evacuation thin and dark brown, but occurring only once or twice in twenty-four hours; the mind dull and slow of comprehension, often wandering when alone, but easily aroused to answer questions correctly; moderate thirst; no appetite; and complains of little or no pain, but much muscular weakness.

Ordered one tea-spoonful of the following emulsion every six hours, viz.: Oil turpentine, ʒij; tinct. opii, ʒj; gum Arabic and white sugar, each ʒiij, rubbed together thoroughly, and added to water ℥ij, the whole well mixed. Also, between each dose of the emulsion, one of the following powders, viz.: ℞ chloride of sodium, ʒj; pulv. opii, 3 grs.; mix and divide into six powders. Allow bread water for drink, with the moderate use of beef tea for nourishment.

15th. Continue same treatment.

16th. General symptoms nearly the same as on admission; has had no evacuation from the bowels, and abdomen seems more full. Ordered a mixture of, castor oil two parts, and oil of turpentine one part, a table-spoonful to be given with six drops of laudanum, and omit all other medicines until it has operated.

17th. 8 o'clock, A.M., the oils taken the previous day had not operated—no material change in the symptoms. Ordered a large enema of warm water containing a table-spoonful of chloride of sodium, to be repeated if necessary until the bowels were freely evacuated. Gave internally spts. nit. dulc. For nourishment, gave beef tea, well salted, freely.

19th. Patient seems more prostrated; tongue drier; pulse more frequent and compressible; mind more dull and wandering. Ordered a powder of carb. ammonia 4 grs., gum camphor 2 grs., pulv. opii ⅓ gr., mixed, every four hours, with animal broth well salted, as freely as the patient would take it.

20th. Symptoms nearly the same; abdomen more tympanitic, and no evacuation from the bowels during the last 48 hours. Ordered a laxative of castor oil and oil turpentine, to be aided, if necessary, by enemas, and after the operation continue the prescription of yesterday.

21st. Morning—patient had three or four copious fæcal evacuations, but more consistent than previously. The abdomen is now less full and tympanitic, the tongue less dry and better color, the skin moist and temperature natural, mind more clear, but pulse 100 and very easily compressed, with a general sense of feebleness. For the first time, also, there is manifested a disposition to cough, with a thick yellowish expectoration. Ordered the following powders, viz.: ℞ sulph. quinine, 12 grs., chloride of sodium, ʒj, pulv. opii, 2 grs., mix, divide into six powders, and give one every four hours. Also, between each of the powders, a tea-spoonful of the following mixture, viz.: ℞ Hive syrup, ℥j, Tinct. opii et camph. ℥j, mixed. Continue free use of animal broth for nourishment.

22d. Continue same treatment.

23d. Patient seems fully convalescent, but feeble. Ordered ℞ carb. ammonia, 18 grs., sulph. quinine, 12 grs., pulv. G. camph., 6 grs., mix, and divide into six powders, one to be taken every eight hours. Continue same diet.

24th. From this time the patient continued steadily to improve, with little other treatment than a regulated but nutritious diet; and was discharged well on the 2nd of the following month.

The foregoing case presents a fair medium between the extreme typhus and the more inflammatory typhoid cases; and the details of treatment must, of course, vary with the varying conditions of each case. But two great leading objects or indications must be kept constantly in view by the practitioner, in treating all the forms of continued fever. The first arises from the essential and primary morbid condition, consisting in a depressed or impaired state of those elementary properties of all the living tissues, called by physiologists *irritability* or susceptibility and *tonicity*. To elevate and sustain these elementary vital properties, and thereby restore a healthy degree of activity to all the organic actions must be a leading object with the rational practitioner. So far as my experience goes, both in hospital and private practice, I have found no remedies more efficient for this purpose, than the chloride of sodium or common salt, given in doses of from 10 to 30 grs., and terebinthinate preparations, aided by the judicious but regular administration of animal broth for nourishment. If in extreme cases more diffusable stimulants are required, I greatly prefer carb. ammonia and camphor, to the alcoholic beverages. Of the 21 cases that recovered out of the 24, here reported, not more than three took either wine or brandy, and those only in small quantities and for a very limited period. The second great object is to guard against the development of local complications, and to remove them when already present. To accomplish these purposes, alterative doses of calomel, anodynes, laxatives, fomentations, counter-irritants, and tepid affusions, may all be required in different cases or even in different stages of the same case. The average period of medical treatment in the twenty-four cases, here included, was about eleven days. The shortest time was four days, and the longest twenty-eight.

Of the twelve cases of dysentery treated during the last term, three only were admitted during the first three days after the attack commenced. All the rest had been laboring under the disease from one to four weeks. Of the two fatal cases, one had been sick, previous to admission, two weeks, during which time he had been under Thompsonian treatment. His tongue, mouth and lips were dry as a chip; the upper lip thin and retracted; the nervous system agitated and restless; the abdomen tympanitic; the discharges small in quantity, consisting of mucus, whitish flocculi, and blood, very frequent and painful; skin was dry and harsh, and the urine very scanty.

He was directed to take a powder composed of calomel 2 grs., pulv. opii 2 grs., every three hours; alternated with a tea-spoonful of the following emulsion, viz.: oil turpentine, ʒij; acetum opii, ʒij; gum Arabic and white sugar, each ʒiij; rubbed together thoroughly, and added to water ℥ij. To aid in allaying the excessive irritability of the rectum, enemas, consisting of cold water four ounces, and tinct. opii a fluid drachm, were to be used occasionally. For nourishment, animal broth.

Under this treatment he improved so rapidly that on the fourth day he left the hospital (without permission, however,) to attend to some item of business, promising the nurse that he would be back in a few hours. He did not return until the lapse of 36 hours, and then with all the symptoms much aggravated. After this no permanent relief was obtained, and he died in about two weeks.

The second fatal case had assumed a chronic form before admission. The patient was much emaciated, feeble, pulse small and frequent; and discharges frequent, painful, and consisting of muco-purulent matter mixed with blood. There was evidently extensive ulceration of the mucous membrane of the colon and rectum. He finally sank exhausted after lingering six weeks, during which he was sustained by stimulants, tonics, anodynes and nourishment. Nit. argenti, both by the stomach and rectum, were tried without benefit; as well as a considerable variety of other medicines.

Of the nine cases of dysentery that recovered, three were admitted during the first three days after the attack. Of these one presented the disease in the ordinary form, characterized by a general febrile condition, active pulse, thirst, frequent and painful mucous discharges, intermixed with blood.

He was ordered calomel and opium, two grains each, every three hours until the pain and discharges were suspended, to be aided, if necessary, by anodyne enemas. Four doses were sufficient to quiet the bowels, and after allowing them to remain so twenty-four hours they were moved by a table-spoonful of castor oil; after which a few doses of the emulsion of oil of turpentine and acetum opii, as given in the last formula, removed the disease entirely, and the patient went out quite well at the end of the first week.

The other two recent cases presented a very different aspect. One was a servant girl, who had been laboring under symptoms of intermittent fever one or two weeks previous to admission. At the time, her expression was dull and heavy; her tongue dry; her pulse small, quick, and feeble; the cheeks and lips of a purplish or livid color; and the discharges frequent, painful and copious, consisting of a reddish serum, much resembling the *lie* from leached ashes. All the symptoms had been much increased at night.

Ordered her a powder, composed of sulph. quinine 4 grs., pulv. opii 2 grs., acetas plumbi 1 gr., mixed, every two hours until six had been taken, unless the discharges were sooner arrested. The next day the symptoms were improved; the discharges less frequent and copious, but not wholly arrested. The powders were continued every four hours, with a teaspoonful of the turpentine emulsion between each dose. The following day a still further improvement had taken place. The powders were given only once in six hours, and the quantity of quinine in each diminished one half. Beef tea, containing a liberal quantity of salt, was given freely from the beginning. Her recovery was rapid, being fully convalescent at the end of the first week after admission.

The other recent case was that of a young man, in whom the dysentery was complicated with well marked cholera symptoms. The discharges yielded to the liberal use of opium and acetate of lead, combined with small doses of calomel; a blister over the epigastrium; and anodyne and astringent injections.

The remaining six cases had all been sick from one to two weeks before admission. Five of them presented a distinct typhoid state, and had taken more or less evacuating medicine. Their treatment consisted chiefly in the administration of the turpentine emulsion with acetum opii, as already given, repeated every four hours, with a powder composed of chloride of sodium 15 grs., and opium 1 gr., between each dose, and the regular use of animal broth for nourishment. Injections of cold water and laudanum were also frequently used, and sometimes a blister over the abdomen.

The sixth case was complicated with intermittent fever, which, together with the dysentery, was promptly removed by the turpentine and opiate emulsion, to each dose of which was added three grains of sulph. quinine.

Chicago, *October* 1, 1852.

